# Are the Physical Environments of Treatment Centres Meeting Recommendations for Patient-Centred Care? Perceptions of Haematological Cancer Patients

**DOI:** 10.3390/ijerph18094892

**Published:** 2021-05-04

**Authors:** Tara Clinton-McHarg, Christine Paul, Rob Sanson-Fisher, Heidi Turon, Michelle Butler, Robert Lindeman

**Affiliations:** 1 School of Medicine and Public Health, The University of Newcastle, Callaghan, NSW 2308, Australia; Chris.Paul@newcastle.edu.au (C.P.); Rob.Sanson-Fisher@newcastle.edu.au (R.S.-F.); Heidi.Turon@newcastle.edu.au (H.T.); 2 Hunter Medical Research Institute, New Lambton Heights, NSW 2305, Australia; 3 School of Psychology, The University of Newcastle, Callaghan, NSW 2308, Australia; 4 Hunter New England Population Health, Wallsend, NSW 2287, Australia; Michelle.Butler@health.nsw.gov.au; 5 Department of Haematology, Prince of Wales Hospital, Randwick, NSW 2031, Australia; Robert.Lindeman@health.nsw.gov.au

**Keywords:** treatment centre environment, physical comfort, wellbeing, hospital design, cancer, haematology

## Abstract

The physical environment of a treatment centre may impact the well-being of patients and their perceptions of care. Outpatients with haematological cancer may be in contact with the treatment centre over long periods and could be particularly affected. This study aimed to identify haematological cancer patients’ perceptions of supportive design elements in the hospital they attended and associations with self-reported mood or well-being. Outpatients from three large metropolitan hospitals in Australia were mailed a self-report questionnaire and responded to statements about the treatment centre concerning their sense of control over the physical surroundings; access to social support; and access to positive distractions. Participants also reported whether they felt the overall environment affected their mood or wellbeing. Of the outpatients who returned the questionnaire (*n* = 165), almost one-quarter (24%) agreed that the physical environment of the hospital affected their mood or well-being. Patients who disagreed that the hospital was a comfortable temperature or agreed that waiting rooms were crowded had significantly higher odds of reporting that the treatment environment affected their mood or wellbeing. Implementing systems to reduce overcrowding in waiting rooms and increasing patient control over personal temperature in clinics may be the most effective strategies to improve patient wellbeing.

## 1. Introduction

Patient-centred care refers to health care that is built around the patient’s perspective of what they want, need, and experience [1,2,3]. Patient-centred care has been acknowledged by the Institute of Medicine (IOM) as one of the six key areas necessary for high-quality health care alongside safety, effectiveness, timeliness, efficiency, and equity [4]. Within the dimension of patient-centred care, the IOM has endorsed six recommendations originally established by the Picker Institute, one of which is to “ensure the physical comfort of the patient” [4]. Physical comfort is a multi-dimensional construct covering three primary themes including pain management, help with activities of daily living, and the hospital environment and surroundings [1].

Patients can experience a range of negative emotions while in a hospital environment, such as stress, anxiety, and uncertainty [5], and commonly undergo procedures that result in short and long-term pain [6]. Reviews of controlled trials suggest that modifying some of the physical attributes of the healthcare environment may lead to reported improvements in the patient experience [7,8,9,10,11]. For example, attributes of the hospital have been associated with a shorter average length of stay [12,13] and significant differences in psychological and physical health outcomes for patients (such as a reduction in stress or pain) [14,15].

A recent qualitative study of patient perceptions of facilitators and barriers to optimal healing post-operatively identified three key needs of patients while in hospital [16]. Firstly, to have a sense of control over aspects of their treatment, such as the ambient features and privacy. Secondly, to have positive distractions and the ability to undertake activities. Thirdly, to be able to access practical and emotional support from family and professionals [16]. The established theory of supportive design similarly recognises that the treatment centre environment can be effective in enhancing patient well-being and reducing patient-reported stress if it enables: (1) perceived control over the physical surroundings; (2) access to social support; and (3) access to positive distractions [17,18,19].

*Perceived control over physical surroundings.* Within the treatment centre environment, individuals may feel a lack of power over their body due to illness, a lack of independence due to the need to rely on others for care, and a lack of control due to the unfamiliarity of their surroundings [20]. Examples of environmental design elements that may influence a patient’s perceived sense of control and autonomy while in the hospital setting include having access to good maps and signs that enable patients to navigate independently [21] or having clean and uncluttered surroundings [22]. Reducing patient exposure to negative sensory features, such as noise [23,24,25,26], odours [27], lack of light [13,28], hard surfaces [29,30], extreme temperatures [31], and extreme colours [32]; being able to escape the clinical environment via access to outdoor spaces, such as gardens and nature [33]; or being able to view the outdoors through large windows [34] may also contribute to increasing a patients’ perceived control over their environment and enhancing patient wellbeing.

*Access to social support.* Access to social support has been shown to reduce patient levels of distress while in the treatment centre environment [35]. This can be facilitated by providing patients with access to private and quiet spaces where they can discuss personal information or express their needs to family, friends, and hospital staff. Studies have also found that arranging furniture so that it optimises acoustic and visual privacy leads to increased wellbeing among patients [36]. Waiting rooms that are not overcrowded or rooms that have space and chairs for family and friends to sit close to the patient are needed for this to occur. Other factors that might also facilitate a patients’ ability to access social support could include being able to connect to WiFi or their mobile network via a mobile phone or the availability of public phones.

*Access to positive distraction.* A positive distraction can be defined as anything that can hold a patient’s attention or interest and leads to positive physiological or psychological changes [18]. Visual distractions within treatment centres might include televisions, reading materials, or the presence of art or images on walls, all of which have been shown to have a positive effect on health and wellbeing [37]. The impact of visual distractions is particularly effective when the object is a real or artificial reproduction of nature [38]. There is a large body of research that demonstrates that exposure to natural elements in a hospital setting (including indoor plants and images portraying realistic nature scenes) has the potential to attract attention and reduce feelings of stress among patients [15,33]. Auditory distractions are also reported to be effective in enhancing patient wellbeing within the hospital setting. A systematic review of 52 trials found that listening to music, and particularly music that was soft, non-lyrical, and comprised mostly of strings and low tones, could have anxiety- and pain-reducing effects for hospital patients [39].

Previous studies have emphasised the importance of assessing the patients’ perspective regarding the physical environment within which they receive treatment, as this is known to mediate reported satisfaction with the quality of health care [40]. A large proportion of this research has focused on the perceptions of inpatients [7]. This is possibly because it is assumed that outpatients will have less contact with the hospital environment compared to inpatients, and therefore supportive design elements will have a limited impact on their wellbeing. However, there are some groups of outpatients who may have extended contact with treatment centre environments over many months or even years. This is true for outpatients who have been diagnosed with haematological cancers such as leukaemias, lymphomas, and myelomas. Many types of haematological cancers, for example, chronic lymphocytic leukaemia (CLL), non-Hodgkin Lymphoma (NHL), and multiple myeloma, can be relapsing and remitting in nature [41]. Outpatient induction chemotherapy and radiation therapy regimens can mean patients might attend the treatment centre multiple times in the same week or in weekly or thrice-weekly cycles for up to six months. While patients are receiving chemotherapy, they are likely to remain in a treatment chair for a few hours. It is therefore critical that the physical comfort of outpatient groups is considered.

Of the small number of research studies that have assessed the physical comfort of the treatment centre from the perspective of cancer outpatients, most focus on a single treatment centre [42] or are qualitative with small samples [43]. Additionally, most research has only focused on one specific design element (e.g., single rooms versus shared rooms), and therefore fails to capture a holistic view of patient perceptions across multiple areas where hospitals may not be meeting patient expectations, and which require improvement.

Given these gaps in the literature, the objective of the current study was to investigate the perceptions of patients receiving outpatient treatment for haematological cancer regarding the presence of treatment centre design elements that are recommended for best-practice, patient-centred care. The specific aims of the study were:

(1) to identify haematological cancer patients’ perceptions regarding the design elements of the treatment centre they currently attended; and
(2) to determine whether there were any associations between patient perceptions of treatment centre design elements and their self-reported mood or well-being related to the overall treatment environment.

## 2. Materials and Methods

Setting: The study sample was recruited from three outpatient haematology clinics, each embedded within large, metropolitan public hospitals located in the capital cities of three different Australian states. These hospitals were not cancer-specific and comprised of a range of departments for complex diseases, surgery, and trauma and emergencies. All of the included clinics exclusively provided outpatient treatment services for both malignant and non-malignant blood disorders. Inpatient treatment for haematological cancer was provided in separate wards within the larger hospital.

Sample: Haematological cancer patients were eligible for the study if they were outpatients at one of the three participating clinics; had attended the clinic more than once; were 18 years of age or older; were able to understand and complete a questionnaire in English; and were at any stage post-diagnosis (e.g., recently diagnosed, currently receiving treatment, receiving follow-up care). Patients were ineligible if they did not have haematological cancer; were considered by their Haematologist to be too unwell (physically or psychologically) to complete the questionnaire; or were attending the clinic for the first time. This last eligibility criterion ensured that participants had sufficient opportunity to experience the clinic’s physical environment prior to completing the questionnaire.

Procedure: Human research ethics approval was gained from the University of Newcastle (H-2010-1324) and all participating treatment centres. At each treatment centre, a Haematologist identified potentially eligible patients from the daily clinic attendance list. A research assistant then approached all eligible patients in the waiting room and invited them to take part in the study. Patients were given a study information sheet, and those who agreed to take part completed a consent form. The gender and age of eligible patients who did not consent to participate were recorded so that any potential bias in the characteristics of the consenting sample could be identified.

Consenting participants completed a 20-min initial survey while waiting for their clinic appointment. The survey collected self-reported information about their demographic characteristics, cancer history and treatment characteristics, psychological well-being (using the Hospital Anxiety and Depression Scale—HADS) [44], and unmet needs (using the Supportive Care Needs Survey Short Form—SCNS-SF34) [45]. Results from this initial survey are reported elsewhere [46,47].

One month after completing the initial survey at the clinic, the research team posted a follow-up paper and pencil survey to each participant to be completed at home. The follow-up survey contained two sections: Section 1 asked for the participants’ perceptions about the physical environment of the treatment centre and Section 2 asked for their perceptions about the safety of the treatment centre [48]. Patients who did not return the survey were sent a reminder letter at two weeks and then another reminder letter at four weeks. Questions regarding the physical environment of the treatment centre were completed at home rather than in the clinic for a number of reasons. First, it was thought that patients may be less inclined to give socially desirable responses when completing these items outside the clinic environment. Second, there was greater potential for the patients to have visited the clinic on a few more occasions prior to completing the follow-up survey so that they had more experiences on which to base their responses.

Measures: Self-reported details regarding the participant’s demographic characteristics, cancer history, and treatment characteristics were collected using the initial questionnaire completed in the clinic waiting room. *Demographic characteristics* included questions about the participants such as the following: gender, age, Aboriginal or Torres Strait Islander identity, marital status, education level, living arrangements, country of birth, employment status, and health insurance status. *Cancer history and treatment characteristics* included questions about the patient’s health status, such as haematological cancer type, stage of cancer (e.g., experiencing the early or late stages of the disease), time since diagnosis, and types of treatments received.

Patients’ perceptions of the treatment centre’s physical environment were collected as part of the follow-up questionnaire. As there was no existing psychometrically robust measure that could be used [49], the Treatment Centre Physical Environment Questionnaire was developed by the research team and based on the Picker Institute’s review of Physical Comfort (one of the eight principles of patient-centred care) [1]. Elements of the physical environment that were reported as important by patients in the review were extracted to form a draft questionnaire. As the data from Picker Institute’s review related to the perceptions of general hospital patients, a subsequent literature review was performed to identify any studies which investigated the physical environment of the treatment centre specifically with cancer patients. Any missing elements of the treatment centre’s physical environment which were identified from the literature review were added to the questionnaire. The draft questionnaire was then reviewed by ten researchers with backgrounds in psychology, behavioural science, haematological cancer, and psycho-oncology. Following this review, the wording of the measure and the response scale was revised, any redundancies were removed, and the reading age of the measure was re-checked. A reading age of 12 years was selected for the measure to ensure that it was suitable for patients who may have only completed primary school-level education.

The resulting questionnaire had 13 items related to perceived control of surroundings, 4 items about social support, and 4 items about positive distractions. Two additional items assessed overall perceptions of the treatment centre (overall, the treatment centre was pleasant and comfortable) and the overall impact of the treatment centre on wellbeing (overall, the physical environment of the treatment centre did not affect my mood or well-being). All 23 items were scored on a 4-point Likert scale from strongly disagree to strongly agree. Seven items were worded in reverse to reduce the risk of response bias (see Table S1).

Statistical analysis: All analyses were conducted in SPSS Version 26.0 [50]. Frequencies and proportions were used to describe the demographic and disease characteristics of participants who returned the Treatment Centre Physical Environment Questionnaire. These demographic and disease characteristics were then compared across the three treatment centres using Chi-square analysis to identify any bias in the clinic samples. The overall perceptions of haematological cancer patients regarding the physical environment of the treatment centre they attended were reported using frequencies and proportions. Univariate analysis (chi-square) was used to identify potential associations between patient perceptions of each treatment centre design element and their self-reported mood or well-being related to the overall treatment environment. Variables identified as having possible associations (*p* < 0.2) in the univariate analyses were included in a logistic regression model with self-reported mood or well-being related to the overall treatment environment as the outcome. Potential clustering of participants by treatment centre was accounted for by adding the variable “centre where treatment was received” to the model. A backwards elimination approach was used with variables *p* > 0.1 removed. Odds Ratios (OR) and 95% confidence intervals (CI) were calculated for significant covariates (*p* < 0.05).

## 3. Results

Across the three clinics, a total of 259 haematological cancer patients were identified as eligible by their Haematologists. Of these, 245 (95%) consented to participate and completed the initial survey in the clinic waiting room. These 245 participants were then mailed the follow-up questionnaire, with 165 (67%) returning the completed Treatment Centre Physical Environment Questionnaire.

### 3.1. Participant Demographic Characteristics, Cancer History, and Treatment Characteristics

The characteristics of participants can be seen in Table 1. The majority of the participants were male (56%), married or had a partner (65%), aged 51–70 (54%), were not employed (63%), and were non-smokers (91%). Differences in the types of haematological cancer that patients had been diagnosed with were observed across centres (χ**^2^** = 30.53, 6, *p* < 0.001). The majority of patients at Centre 1 had been diagnosed with lymphoma (42%), whereas the majority of patients at Centre 2 and Centre 3 had leukaemia (50% and 53% respectively). Centre 2 also had a higher proportion of patients with myeloma (34%) compared to the other two sites. These differences in cancer types may also relate to differences in the age of participants observed across the three centres (χ**^2^** = 41.28, 6, *p* < 0.001), as different haematological cancers are more common among certain age groups [41]. At Centre 2, a higher proportion of younger patients aged 31–50 (38%) completed the survey, whereas at Centres 1 and 3, a higher proportion of patients 71 years or older participated (32% and 36% respectively). There were no other significant differences between the characteristics of the three centre samples.

### 3.2. Perceptions Regarding the Presence of Supportive Design Elements at the Treatment Centre

Patient agreement regarding the presence of supportive design elements at the treatment centres can be seen in Table 2, Table 3 and Table 4. Across all three sites, eighty percent or more participants agreed that the centre they attended was clean (94%, *n* = 153), had no peeling paint or cracks (86%, *n* = 140), had maps and signs (85%, *n* = 137), was free from odours (84%, *n* = 136), had comfortable furniture (83%, *n* = 135), had good phone access (83%, *n* = 129), was a comfortable temperature (81%, *n* = 133), had windows and natural light (80%, *n* = 132), and had uncrowded waiting rooms (80%, *n* = 129). A high proportion of participants agreed that positive distractions such as television and reading materials were available (89%, *n* = 144). The majority of participants (89%, *n* = 143) agreed that “overall, the treatment clinic was pleasant and comfortable”.

The presence of other types of distractions was reported to be much lower, with 77% (*n* = 119) disagreeing there was relaxing music to listen to, 69% (*n* = 108) disagreeing there were indoor plants, and 32% (*n* = 52) disagreeing there was art or images on the walls. More than one-third of participants perceived that the treatment centre did not have private spaces for them to use (37%, *n* = 57), was cluttered (35%, *n* = 58), and that the colour scheme was dull and dreary (34%, *n* = 54).

Almost one-quarter of participants (24%, *n* = 39) agreed that the overall physical environment of the treatment centre affected their mood or well-being.

### 3.3. Associations between Perceptions of Supportive Design Elements and Mood or Wellbeing Being Affected by the Treatment Environment

Following chi-square analysis, perceptions of 13 supportive design variables were identified as having possible associations (*p* < 0.2) with the patient-reported agreement that the physical environment of the treatment centre affected their mood or well-being. These 13 variables, along with the variable centre where treatment was received, were added to the logistic regression model. The results of the final logistic regression model can be seen in Table 5. Two variables had *p*-values less than 0.05 and confidence intervals that did not contain 1.

Patients who disagreed that the hospital was a comfortable temperature had four times the odds of agreeing that the treatment environment affected their mood or wellbeing (OR 4.02, [1.50, 10.75]), while patients who disagreed that waiting rooms were not crowded had three times the odds of agreeing that the treatment environment affected their mood or wellbeing (OR 3.46, [1.35–8.84]).

## 4. Discussion

This is one of the first studies internationally to quantitatively assess haematological cancer outpatients’ perceptions of the supportive design elements of the treatment centre they attend. While almost 90% of participants agreed that the overall environment of the centre was pleasant and comfortable, there were some elements of the physical environment that patients perceived were not present. Additionally, almost one in four participants agreed that the overall treatment environment affected their mood or well-being.

*Increase a sense of control over surroundings.* More than one-third of outpatients in the current study perceived that the treatment clinic environment was cluttered. Previous studies conducted with non-cancer populations identified that clutter in hospital corridors was the primary element that impeded independence [51]. Qualitative interviews conducted with medical unit patients who had dementia in a large hospital in Canada revealed that clutter due to linen carts, beds, and medical equipment blocking hallways created physical obstacles which impacted patient safety. Multiple signs and posters also created visual noise which was reported to confuse and distress patients [51]. People who were less mobile, such as wheelchair-users or those with visual impairment, found cluttered environments particularly difficult to navigate [51]. Removing clutter within hospitals has been acknowledged in other studies as important to increase patient’s perceived control over their environment and enable successful wayfinding, especially for older adults [52,53]. Controlling the amount of signage, increasing storage options, or providing discrete screening should be considered to reduce the impact of physical obstacles and visual noise for patients [54].

Over one-third of outpatients in the current study perceived the colours in the hospital environment to be dull and dreary. A previous study of hospital environments (dull versus bright, boring versus stimulating, etc.) assessed the impact of a newly designed waiting room on patient’s environmental appraisals [55]. Two samples of neurology outpatients were compared: those who attended the existing traditional waiting room and those who attended the newly designed waiting room. A significant difference in patient appraisals between groups was found, with the new environment rated more “colourful, positive, stimulating, attractive, relaxed, comfortable, cheerful, good, lively, bright, motivating, pleasant, and open” [55]. Ratings of the newly designed room were associated with a significant decrease in self-reported stress over time compared to the traditional waiting room where ratings were associated with an increase in stress [55]. Given that colour is a simple element to alter, it should be considered as a minimal intervention to address patient comfort.

*Increase access to social support.* Almost 40% of participants in the current study perceived there was a lack of private spaces that could be accessed by patients. The preferences for different types of spaces when receiving treatment for cancer have previously been explored. In a treatment centre in Louisiana, patients receiving infusion chemotherapy were asked about their preferences for private, semi-private, or open-treatment spaces. Patients who preferred private spaces listed wanting to nap, social interaction, and escaping noise as some of the reasons that private spaces were desirable [56]. In a second study conducted in the same centre, the research team observed 252 infusion patients and found that those in private spaces were more likely to have family members or friends with them (66%) compared to patients in open spaces (58%) [56]. These findings support the idea that private spaces may enable outpatient access to social support. However, the research also reports that patients still valued the patient-to-patient interaction provided by semi-open and open spaces [56]. Having retractable screens that patients can close for privacy and open when they desire broader interactions may be one way to increase patients’ physical comfort without requiring substantial building redesign [56].

*Increase access to positive distractions.* A large proportion of outpatients in the study disagreed that positive distractions such as relaxing music, plants, and artworks were present. A Cochrane review summarised the evidence regarding the psychological and physical impact that music can have on patients with cancer. The meta-analyses of 13 studies measuring anxiety and 7 studies measuring pain confirmed that music could have a beneficial impact on these outcomes [39]. The effectiveness of plants as a distraction for patients has been demonstrated in a trial where patients were randomly assigned to identical rooms which had no plants (control) or multiple plants (intervention) [15]. Patients in the intervention group had significantly lower ratings of pain and anxiety and significantly higher ratings of satisfaction and positive appraisals of the hospital environment [15]. Another randomised controlled trial demonstrated that cancer patients having bone marrow aspirates and biopsies were significantly more likely to report only mild pain if they were able to view an image of nature compared to patients with no nature image present [38]. The known benefits of music, plants, and nature images and the potentially low cost to implement these changes in cancer outpatient clinics provide additional opportunity to improve the physical comfort of patients.

*Address design elements that are associated with mood and wellbeing.* Patients who disagreed that the temperature in the clinic was comfortable had much higher odds of reporting that the treatment environment affected their mood or wellbeing compared to those who agreed the temperature was comfortable. This is not surprising, as a previous study with 300 cancer patients from five cancer centres in the United States had similar findings. When asked about the hospital environment they desired when receiving outpatient infusion treatment, temperature control was ranked as the top priority [57]. Cancer patients receiving treatment infusions are often exposed to chilled fluids for many hours. Systematic reviews of trials conducted in hospitals have shown that experiencing a low core body temperature (below 36 degrees Celsius) can impact tissue healing and immune function [58,59]. Increased thermal comfort has also been associated with reductions in patient anxiety [60]. It is acknowledged that thermal comfort can be difficult to achieve in health settings due to the threat of bacteria growth and the different temperature needs of staff compared to patients [31]. For this reason, it is recommended that hospitals invest in providing specific heating, ventilation, and air conditioning zones in order to improve patient mood and wellbeing [31]. Other ways to improve thermal comfort include installing overhead radiant heaters or having chairs that can be heated while patients receive outpatient care [57].

Patients who perceived that the clinic waiting room was crowded had much higher odds of reporting that the treatment environment affected their mood or wellbeing compared to those who did not think it was crowded. A crowded waiting room may impact the physical comfort of patients in a number of ways, including frustration from being unable to find a seat, pain from standing, loss of social support if unable to sit with family or friends, and the ambient impact of noise levels. Having an overcrowded waiting room also makes it more likely that patients could be exposed to situations that cause them emotional distress. In a study of 355 outpatients in an oncology clinic, 85% reported that waiting had an emotional cost, 35% reported being upset by talking about their illness to other patients in the waiting room, and 25% reported being upset by seeing other sick people [61]. The inability to escape a crowded waiting room due to fear of missing their appointment might also contribute to a patient’s perceived wellbeing. In a study of almost 700 cancer patients, it was found that using a pager system that buzzed to alert patients when it was time for their appointment contributed to increased patient satisfaction [42]. Similar results were found in another study with 78% of patients reporting they would have liked to have been given a buzzer that called them back at the time of their appointment [61]. This type of system could enable patients to leave a crowded waiting room without fear of missing their appointment and potentially increase their perceived sense of control over the hospital environment.

Future Research Directions. The findings from the current study support existing literature regarding the role that treatment centres may play in the healing process and highlight aspects of the physical hospital environment that could be further considered to enhance the physical and mental wellbeing of cancer patients. Providing recommended, evidence-based design elements in oncology clinics has been shown to improve cancer patients’ overall satisfaction with care [42]. Including both outpatients and inpatients in co-design processes to create patient-centred hospitals is recommended, given that some groups of outpatients (such as those with haematological cancer) can have extended contact with the treatment centre environment. Additionally, patients with cancer can be receiving many different approaches to therapy dependent on their stage of disease (e.g., newly diagnosed, relapsing disease, receiving palliative treatment), which may alter their needs within the hospital environment. It is important that patients who are at different stages of the cancer journey are also able to contribute to the design process.

When assessing the supportive design elements of treatment centres in future studies, a number of different methodological approaches should be considered to increase the sophistication of the detail that can be reported. For example, in the current study, patients completed the survey at home and were recalling overall perceptions of the hospital environment, and rather perceptions at a specific date or time. This means there was no way to objectively measure some elements of the environment (for example, conditions important for thermal comfort such as relative humidity or radiant temperature). Future studies could consider collecting patient perceptions of hospital environments concurrently with objective assessments of environmental elements to assist with interpretation and decision-making. Specific details of the treatment centres that patients attended were unable to be reported in the current study (for example the names of centres and descriptions/images of hospital features that would identify them), as hospitals did not provide specific consent to be identified as part of the research. However, the ability to include more context and descriptions of design elements explored, such as specific colours, materials, and dimensions, should be considered in further research. Allowing patients to provide ratings on the perceived quality of design elements, in addition to the presence or absence of such elements, is also desirable and would increase the relevance of research findings to collaborators from a variety of disciplines including healthcare and the built environment.

Limitations. The current study has a number of limitations which may impact the generalisability of these findings. The study took place in the context of outpatient haematology clinics located within large, publicly funded metropolitan hospitals in Australia. Therefore, the results may not translate to other outpatient samples or clinical settings. Also, less than 5% of participants were aged 30 years or younger, and less than half were women; therefore, the reported perceptions of patients in the study may not be reflecting the views of these groups. However, the three participating hospitals were in three different major cities and states in Australia, and the sample size was large, which helps to broaden the representation of outpatient haematological cancer patients. A further limitation is that participants completed the survey while at home rather than in the treatment centre setting. This location was chosen as it was thought that patients may be less inclined to give socially desirable responses outside the clinic environment. However, it may also have meant that patient answers were affected by recall bias. The survey used in the study was developed by the research team, as no psychometrically robust measure exists [49]; however, published literature was used to guide item development so that all potentially important evidence-based design elements for hospitals were covered. Additionally, wellbeing and mood related to the overall treatment environment were self-reported and not measured using a standardised tool of depression or anxiety. This was done to avoid overburdening participants. Future research could consider using more standardised measures of mood to investigate associations with patient perceptions of the treatment environment. Finally, it should be noted when interpreting the results that the risk of Type 1 error was high due to the number of univariate comparisons. Therefore, only associations identified from the subsequent multivariate analyses (logistic regression) are discussed.

## 5. Conclusions

The current research provides useful information regarding an important aspect of patient-centred care for haematological cancer patients. The findings can help to inform health services regarding elements of the treatment centre environment that could be addressed to enhance the physical comfort of outpatients with blood cancers who may have extended contact with hospital clinics. Identifying ways to increase patient control over the temperature in the clinic to improve thermal comfort and utilising technology or other systems that could help to reduce overcrowding might be most effective for improving patient mood and wellbeing. Other minimal, low-cost interventions, such as adding colour, plants, and nature images; providing screening for privacy; and reducing clutter in waiting rooms and corridors, could also be considered as patients currently perceive these elements to be lacking.

## Figures and Tables

**Table 1:** Socio-demographic and disease characteristics of the study sample (*n* = 165).

Characteristic		*n* (%)
Gender	Male	93 (56)
	Female	72 (44)
Age	≤30	7 (4.3)
	31–50	33 (20)
	51–70	89 (54)
	≥71	35 (21)
Type of cancer	Leukaemia	72 (44)
	Lymphoma	47 (29)
	Myeloma	35 (21)
	Other blood cancer	10 (6.1)
Time since diagnosis	<2 years	88 (54)
	2+ years	75 (46)
Stage of cancer	Early	42 (27)
	Advanced	47 (30)
	In remission	14 (8.9)
	Don’t know	54 (34)
Treatment received	Chemotherapy and other treatment	110 (69)
	Chemotherapy only	33 (21)
	Other treatment only (no chemotherapy)	9 (5.7)
	No treatment	7 (4.4)
Marital status	Married or partner	107 (65)
	Single, divorced, widowed	57 (35)
Education completed	High school or below	69 (43)
	Trade/ vocational training	48 (30)
	University degree	37 (23)
	Other	8 (4.9)
Employment status	Currently employed	60 (37)
	Not employed	102 (63)
Place of birth	Australia	121 (74)
	Other	43 (26)
Smoking status	Current non-smoker	150 (91)
	Current smoker	14 (8.5)
Place treatment was received	Centre 1	93 (56)
	Centre 2	58 (35)
	Centre 3	14 (8.5)

**Table 2:** Patient perceptions of hospital elements related to the Perceived Control of Surroundings and univariate associations with self-reported mood or well-being related to the overall treatment environment (*n* = 165).

		Overall Affected Mood or Wellbeing		
		Disagreed	Agreed	
Perceived Control of Surroundings	Present at the Centre	*n* (%)	*n* (%)	*p*-Value
The hospital had maps/signs to help you find your way around	Disagreed	16 (64)	9 (36)	0.129
	Agreed	107 (78)	30 (22)	
The hospital had clocks in waiting rooms	Disagreed	30 (67)	15 (33)	0.098
	Agreed	81 (79)	21 (21)	
The hospital had plenty of windows and natural light	Disagreed	22 (69)	10 (31)	0.269
	Agreed	103 (78)	29 (22)	
The hospital had outdoor spaces	Disagreed	28 (76)	9 (24)	0.934
	Agreed	90 (75)	30 (25)	
The hospital was clean	Disagreed	6 (60)	4 (40)	0.253 ^a^
	Agreed	118 (77)	35 (23)	
The hospital was free from clutter	Disagreed	41 (71)	17 (29)	0.231
	Agreed	83 (79)	22 (21)	
The hospital did not have cracks in walls or peeling paint	Disagreed	12 (54)	10 (46)	0.017
	Agreed	111 (79)	29 (21)	
The hospital was quiet	Disagreed	22 (58)	16 (42)	0.003
	Agreed	100 (81)	23 (19)	
The hospital was free from odours	Disagreed	15 (58)	11 (42)	0.013
	Agreed	109 (80)	27 (20)	
The hospital had comfortable furniture	Disagreed	13 (48)	14 (52)	<0.001
	Agreed	110 (81)	25 (19)	
The hospital was a comfortable temperature	Disagreed	17 (55)	14 (45)	0.002
	Agreed	108 (81)	25 (19)	
The colour scheme of the hospital was calm and relaxing	Disagreed	25 (57)	19 (43)	<0.001
	Agreed	98 (84)	18 (16)	
The colour scheme of the hospital was not dull and dreary	Disagreed	32 (59)	22 (41)	<0.001
	Agreed	88 (85)	16 (15)	

Note: The sample size is not *n* = 165 for all variables due to missing data. ^a^ Fishers exact test as expected cell count < 5.

**Table 3:** Patient perceptions of hospital elements related to Access to Social Support and univariate associations with self-reported mood or well-being related to the overall treatment environment (*n* = 165).

		Overall Affected Mood or Wellbeing		
		Disagreed	Agreed	
Access to Social Support	Present at the Centre	n (%)	n (%)	*p*-Value
The hospital had good phone reception and public phones	Disagreed	17 (63)	10 (37)	0.112
	Agreed	100 (78)	29 (22)	
The hospital had uncrowded waiting rooms	Disagreed	18 (56)	14 (44)	0.003
	Agreed	105 (81)	24 (19)	
The hospital had quiet spaces	Disagreed	22 (55)	18 (45)	<0.001
	Agreed	99 (82)	21 (18)	
The hospital had private spaces	Disagreed	41 (72)	16 (28)	0.524
	Agreed	75 (76)	23 (24)	

Note: The sample size is not *n* = 165 for all variables due to missing data.

**Table 4:** Patient perceptions of hospital elements related to Positive Distractions and univariate associations with self-reported mood or well-being related to the overall treatment environment (*n* = 165).

		Overall Affected Mood or Wellbeing		
		Disagreed	Agreed	
Positive Distractions	Present at the Centre	n (%)	n (%)	*p*-Value
The hospital had art, photography, or other images on walls	Disagreed	36 (69)	16 (31)	0.181
	Agreed	86 (79)	23 (21)	
The hospital had indoor plants	Disagreed	78 (72)	30 (28)	0.206
	Agreed	40 (82)	9 (18)	
The hospital had television and reading materials	Disagreed	13 (72)	5 (28)	0.771 ^a^
	Agreed	110 (76)	34 (24)	
The hospital had relaxing music to listen to	Disagreed	89 (75)	30 (25)	0.715
	Agreed	28(78)	8 (22)	

Note: The sample size is not *n* = 165 for all variables due to missing data. ^a^ Fishers exact test as expected cell count < 5.

**Table 5:** Final logistic regression model of elements of the hospital that were associated with mood or wellbeing being affected by the treatment environment.

Variable		Odds Ratio (95% CI)	*p*-Value
The hospital was a comfortable temperature	Disagreed	4.02 (1.50–10.75)	0.006
	Agreed	Reference	
The hospital had uncrowded waiting rooms	Disagreed	3.46 (1.35–8.84)	0.010
	Agreed	Reference	

## Data Availability

Data supporting reported results can be requested from the corresponding author.

## Supplementary Materials

The following are available online at https://www.mdpi.com/article/10.3390/ijerph18094892/s1, Table S1: Copy of the Treatment Centre Physical Environment Questionnaire.
