# Supplementary material for: Are the Physical Environments of Treatment Centres Meeting Recommendations for Patient-Centred Care? Perceptions of Haematological Cancer Patients
Source: Int J Environ Res Public Health. 2021 May 4;18(9):4892. doi: 10.3390/ijerph18094892 (PMC8125011; doi:10.3390/ijerph18094892)
Supplement: Supplementary file 1 [file ijerph-18-04892-s001.zip › ijerph-1166860-supplementary.pdf]

Table S1: Copy of the Treatment Centre Physical Environment Questionnaire

We would like to know your views about the physical environment of the hospital where you received most of your cancer treatment.

Please circle how much you agree or disagree with the following statements:		Strongly Disagree	Disagree	Agree	Strongly Agree
**The hospital was:**					
**1.**	Dirty	1	2	3	4
**2.**	Free from clutter	1	2	3	4
**3.**	Noisy	1	2	3	4
**4**	A comfortable temperature	1	2	3	4
**5.**	Free from bad smells	1	2	3	4
**The hospital had:**					
**6.**	Overcrowded waiting rooms	1	2	3	4
**7.**	Clocks in the waiting rooms	1	2	3	4
**8.**	Plenty of windows and natural light	1	2	3	4
**9.**	Cracks in the walls or peeling paint	1	2	3	4
**10.**	Comfortable furniture	1	2	3	4
**11.**	Art and photography, or other images on the walls	1	2	3	4
**12.**	Indoor plants	1	2	3	4
**13.**	Maps and signs to help you find your way around	1	2	3	4
**14.**	Good mobile phone reception and public phones	1	2	3	4
**The colour scheme of the hospital was:**					
**15.**	Dull and dreary	1	2	3	4
**16.**	Calm and relaxing	1	2	3	4
**The hospital had:**					
**17.**	Quiet spaces	1	2	3	4
**18.**	No outdoor spaces	1	2	3	4
**19.**	Private spaces	1	2	3	4
**20.**	No entertainment e.g. TV or reading materials	1	2	3	4
**21.**	Relaxing music to listen to	1	2	3	4
**Overall, I felt the hospital environment:**					
**22.**	Was pleasant and comfortable	1	2	3	4
**23.**	Did not affect my mood or wellbeing	1	2	3	4
